# Early post-fire declines of mammals in a tree-and-shrub savanna

**DOI:** 10.1007/s00442-026-05939-w

**Published:** 2026-07-23

**Authors:** Stijn Verschueren, Maria Laura Ruozzi, Laurie Marker, Herwig Leirs, Bogdan Cristescu

**Affiliations:** 1 Cheetah Conservation Fund, Otjiwarongo, Namibia; 2 Division of Conservation Biology, University of Bern, Bern, Switzerland; 3 Evolutionary Ecology group, Department of Biology, University of Antwerp, Antwerp, Belgium; 4 School of Agriculture and Natural Resources Sciences, Namibia University of Science and Technology, Windhoek, Namibia; 5 Centre for Environment and Society & School of Applied Sciences, University of Brighton, Brighton, UK

**Keywords:** Community dynamics, Disturbance, Ecological succession, Fire ecology, Habitat change, Kalahari, Occupancy modelling

## Abstract

Wildfires strongly shape savanna ecosystems through vegetation restructuring, nutrient recycling and abiotic change. Post-fire habitat conditions can progressively redistribute wildlife, with differential responses between functional groups and across vegetation successional stages. Using camera traps and environmental data loggers, we monitored a large landscape (650km^2^) affected by a wildfire in a tree-and-shrub savanna of eastern Namibia to quantify early-phase responses of medium- to large-size mammals across burned areas, adjacent proximal zones and unburned controls. We applied a multi-season, multi-species occupancy model to six months of camera-trap data spanning one month pre-fire and five months post-fire to assess community- and functional group–level dynamics of 31 species. Burned areas experienced higher temperatures, lower humidity, and reduced vegetation growth following the wildfire. Community-level mammal occupancy declined progressively in burned areas (from 0.53 to 0.33), while remaining nearly stable in control areas (from 0.45 to 0.43) and showing small declines in proximal zones (from 0.58 to 0.52). Functional group responses differed in intensity: small carnivores and burrow-associated species showed the strongest fire-driven declines, followed by large carnivores, browsers, and grazers. Proximal areas did not function as refugia and instead showed slight occupancy declines relative to controls, indicating that fire impacts extended beyond the burned perimeter. Our findings demonstrate that drastic habitat change in the first months after a wildfire drives landscape-scale redistribution of savanna mammals, with the strongest effects on small-bodied and specialist species. These results underscore the importance of maintaining large, well-connected habitats to allow adequate space and time for recovery between fire events.

**Supplementary Information:**

The online version contains supplementary material available at 10.1007/s00442-026-05939-w.

## Introduction

Wildfires are major structuring forces in savanna ecosystems, rapidly altering vegetation, soil properties, and microclimate across vast spatial scales (Bond and Keeley 2005). By removing organic matter and acting as a mineralizing agent, fires accelerate nutrient release in soils and trigger vegetation succession that reshapes landscape-level habitat heterogeneity (Pausas and Bond 2020). The savanna biome has co-evolved with frequent fires, maintaining a landscape mosaic of grasslands, shrublands and woodlands that supports high faunal diversity (Simpson et al. 2022). This fire-driven heterogeneity is critical for maintaining wildlife populations and ecosystem processes, while also supporting millions of rural livelihoods, particularly across Africa (Pausas and Keeley 2019). However, altered fire regimes alongside woody plant encroachment, climate change and megaherbivore depletion are disrupting the natural dynamics that sustains these systems (Shen et al. 2025).

Monitoring wildlife responses to wildfires is important for understanding savanna ecosystems, especially under changing fire regimes (Sergio et al. 2018). Herbivore responses to wildfires have been well-studied and generally show attraction to recently burned areas due to nutrient-rich vegetation regrowth and reduced predation risk (Archibald et al. 2005; Eby et al. 2014). On the other hand, carnivore responses are less understood, but predators may track prey into burned areas, avoid burned areas due to loss of cover for ambush hunting, or show neutral responses driven by their wide-ranging behavior (Geary et al. 2020; Gigliotti et al. 2022). These patterns typically emerge over multiple months to years after the disturbance, after initial recovery has begun (Doherty et al. 2022).

In contrast, the early post-fire period is characterized by strong modification of the local environment, including elevated temperatures, reduced humidity, loss of structural vegetation, and suppressed resource availability that persist for weeks to months depending on fire severity, ecosystem type and post-fire rainfall (Zahed and Bączek-Kwinta 2023). During this critical window, wildlife may progressively abandon fire-affected areas due to direct physiological stress, increased thermoregulatory costs and degraded foraging conditions. Understanding these early-phase dynamics is essential because cumulative impacts on occupancy over shortened fire-return intervals can amplify the vulnerability of already-stressed populations and lead to local extinctions.

We used a natural experiment in a tree-and-shrub savanna landscape in eastern Namibia, where a large wildfire in October 2021 affected a substantial portion of a camera-trap study landscape. Using an established monitoring network with camera traps and abiotic sensors, we recorded temperature and relative humidity, and tracked occupancy of medium- to large-sized mammals over a six-month period (one month pre-fire baseline and five months post-fire), across three landscape zones: burned areas, proximal areas and unburned control areas.

We hypothesize that in the early post-fire period, burned areas become progressively less suitable for wildlife due to abrupt habitat changes. We predict that occupancy will decline in burned areas, while control areas remain stable, or experience broader environmental changes such as in response to seasonal resource availability. We also predict that occupancy trends in burned and proximal areas will diverge from control area trends in opposite directions: burned areas showing steeper declines than controls, while proximal areas show either increases or smaller declines. Such divergence would signal that proximal areas function as refugia, where wildlife is displaced from burned areas to adjacent zones.

While the community trends across the three landscape zones form our central predictions, we further hypothesize that responses will vary across functional groups based on their ecological requirements: Grazers, which rely on continuous grass layers, are expected to show the steepest declines in burned areas due to the immediate loss of forage (Fuhlendorf et al. 2009). Browsers may exhibit intermediate responses as fire affects cover, but their reliance on more fire-resistant woody vegetation may allow for persistence in unburned patches (Levick et al. 2009). Large carnivores are predicted to show variable responses as prey density may decrease, but their wide-ranging behavior and ability to access prey across multiple landscape zones may buffer them against local habitat changes (Gigliotti et al. 2022). Small generalist carnivores may be less constrained by prey availability due to dietary breadth, unless the habitat changes compromise hunting efficiency or reduce activity of small prey species across multiple prey types (Schuette et al. 2014). Small specialist carnivores will likely decline due to their strong dependence on specific microhabitat features and prey bases that are directly compromised by fire (Hale et al. 2022). Burrow-users are hypothesized to persist in the short term due to subterranean thermal refugia (Linley et al. 2024), though their continued occupancy will depend on the post-fire availability of vegetative cover and foraging resources.

## Methods

### Study area

We conducted the study in the Central Kalahari savanna system of Omaheke region (S21.810271, E18.369111), eastern Namibia. The area experiences a unimodal rainfall pattern averaging 350 mm annually, with the dry season spanning from May to September, and the wet season from October to April with most rainfall falling in January and February (Atlas of Namibia Team 2022). The study area covers several extensive privately-owned farms used for free-range livestock production and/or wildlife hunting, and permission to access them was provided by the landowners. In Namibia, privately-owned farms comprise 44% of the total land of the country, and it has been estimated that approximately 80% of the larger wildlife species are located on private farms, therefore outside of protected areas (Atlas of Namibia Team 2022).

**Fig. 1:**
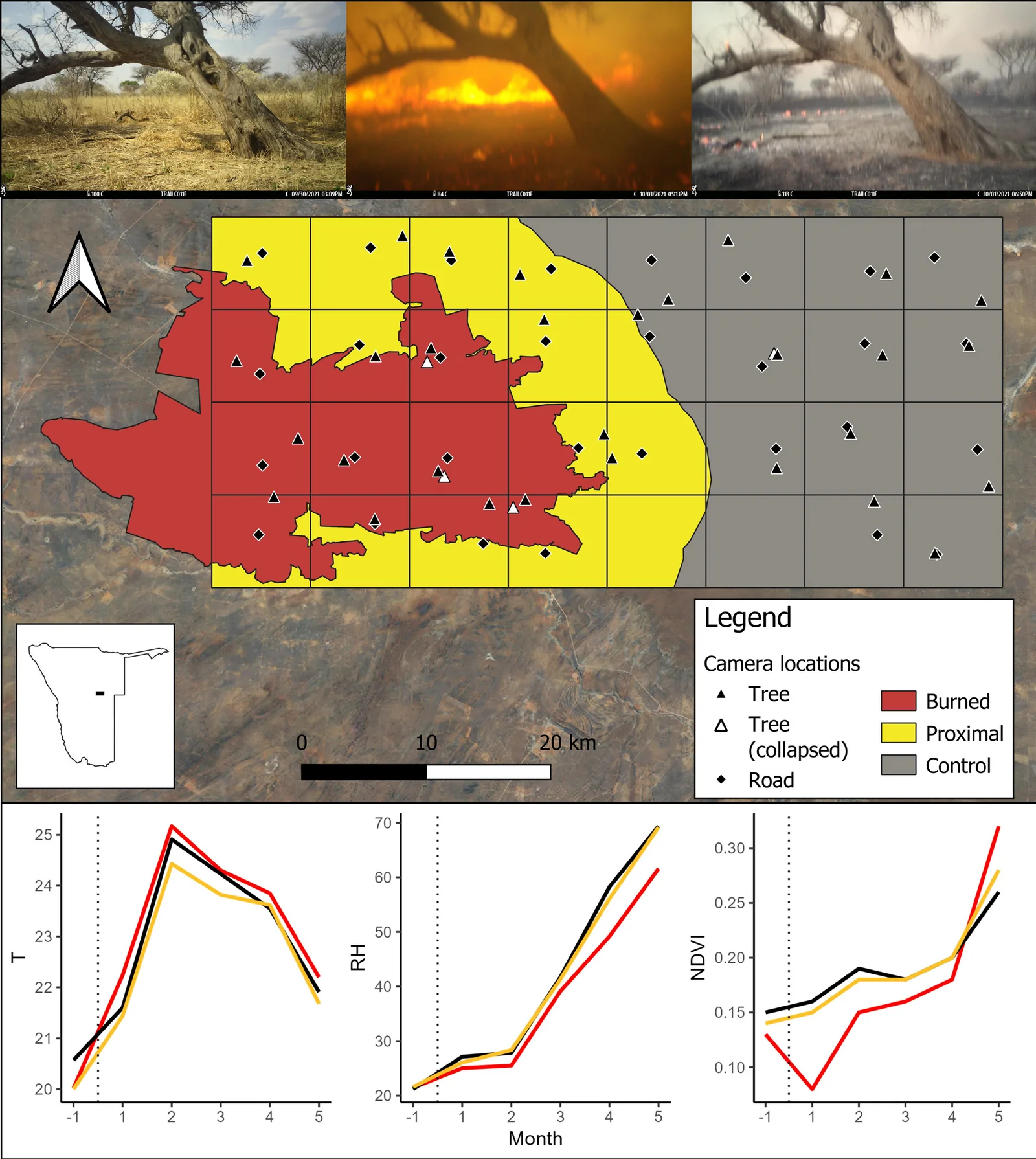
Study area map (middle) displaying camera trap locations, study grid and landscape categorization into burned, control and proximal areas. Top panel plots are series of camera trap images at one marking site before, during and after the wildfire. Bottom panel plots show temperature in degrees Celsius (T), relative humidity in percentages (RH) and the Normalized Difference Vegetation Index (NDVI) over time with the time of the wildfire (1–4 October 2021) denoted with a vertical dotted line. Line colors correspond to landscape categorization

Between 1 and 4 October 2021, a wildfire burned approximately 650 km^2^ of savanna habitat, which by chance covered about a third of the area that was being monitored by camera traps (Figs. 1, S1 & S2). The camera trap survey initially aimed to understand carnivore community dynamics within the system, yet the unforeseen fire disturbance represented a unique opportunity to conduct this study focusing on the responses of medium- to large-sized mammals to the disturbance. The wildfire occurred late in the dry season, hence our post-fire study period captured wildlife response during the onset of the rainy season.

We also acknowledge that livestock, mostly cattle, were removed from the burned areas after the wildfire, with post-fire vegetation management being passive and focused on allowing natural grass regeneration following rainfall until sufficient seed set and dispersal had occurred to restore the seed bank, after which livestock were returned to the area. This recovery period extended beyond our five-month post-fire study window, so livestock grazing pressure was absent from the burned sites throughout the sampling period.

Because wildlife respond to livestock presence, our study may have captured responses to both the fire disturbance and the temporary exclusion of cattle. The guilds most likely to interact with cattle are large grazers and to a lesser extent browsers, which compete with livestock for forage, and large carnivores, which may use cattle as an opportunistic prey resource. In the Discussion, we explain why fire is the more likely primary driver of the observed wildlife responses.

### Data collection

From August 2021 until March 2022, a total of 64 camera trap stations were deployed across the study area, which spanned 30 grid cells and covered approximately 2048 km² (8 km × 8 km per grid cell). In each grid cell, we deployed one camera trap station within 2 km of the cell centroid, positioned alongside a private farm dirt road where a game trail intersected. In addition, we deployed one camera trap station at a carnivore (i.e. cheetah) marking site identified by a prior track survey effort supported with a scat detection dog (Fig S1) (Verschueren et al. 2025). Cheetah marking sites typically are located at trees with distinct characteristics (Walker et al. 2016) and are often used by other carnivores especially leopards (Verschueren et al. 2023). Two grid cells were omitted because of access restrictions.

Each camera trap station had two camera traps placed opposite of each other. In addition, we placed a data logger that recorded temperature and relative humidity at each marking site. Camera traps (Browning Strike Force Pro XD black flash) and data loggers (HOBO ONSET MX2301A) were placed approximately 60 cm above the ground. Camera traps placed at roads were programmed to take a burst of five images per trigger, and triggers were separated by five minutes. Camera traps placed at marking sites were programmed to take a burst of three images per trigger, and triggers were separated by one minute. Data loggers were programmed to record meteorological information every 5 min. Camera traps and data loggers were active for a minimum of 7 months and serviced for functionality every 30–60 days. The use of two cameras per station, combined with regular maintenance visits, ensured continuous camera-trap monitoring with no periods of inactivity, except immediately following the fire (see below).

Camera-trap stations were spaced on average 1.9 km from their nearest neighbor (± 1.15 km SD; range 0.18–4.75 km). At six grid cells, the road and marking tree stations fell within one kilometer of each other and we explored whether this clustering induced spatial autocorrelation by refitting the model with either station of each pair removed. This did not affect the model outcomes and therefore we retained both stations in the final model. Model outputs were interpreted as the intensity of local site use rather than occupancy in a strict sense, to keep the inference relevant across the full suite of species detected.

Following the fire event, we divided the study area into three landscape zones: the burned area (B) where the fire occurred, the proximal area (P) as an 8 km buffer surrounding the burned area, and the control area (C) as an area beyond 8 km that remained unaffected by the wildfire (Fig. 1). The buffer size was based on the average ranging size of medium- to large sized mammals (Van der Weyde et al. 2018) and was designed to investigate whether the proximal area was used differently compared to the burned and the control area. Based on the wildfire spread pattern, our sampling grid contained by chance 17 camera trap stations in the burned area, 17 in the proximal area and 26 in the control area. In the month of the fire, only 11 camera trap stations remained active in the burned area after losing 6 to the fire conditions. These were replaced from the first month after the fire and remained active afterwards. Three trees in the burned area and one tree in the control area collapsed and camera traps were redeployed at a near-by marking tree.

### Abiotic conditions and vegetation change

For each of the three zones, we calculated monthly averages for relative humidity and temperature based on the datalogger information. Similarly, we calculated the Normalized Difference Vegetation Index (NDVI) to assess changes in vegetation productivity over time (Pettorelli et al. 2005). To represent the immediate habitat conditions available to medium- and large-sized mammal species, we created a buffer with 2 km radius around each camera trap location for which we calculated NDVI values (Verschueren et al. 2021). This was done on a monthly basis and we averaged NDVI values across landscape zones. Temporal changes in abiotic and vegetation parameters within the control zone were attributed to seasonal variation, whereas divergences observed in the burned areas were interpreted as effects of the fire on local environmental conditions.

### Species groups and detections

Camera trap images were classified to species-level using TrapTagger, an open-source web application that uses artificial intelligence in combination with a manual annotation interface to process camera trap data (WildEye 2023). Independent captures were separated by a 30-min interval threshold.

We included 31 medium- to large-sized mammal species (> 1 kg) that were assigned to six functional groups: grazers, browsers, burrow-users, large carnivores, small carnivore generalists and small carnivore specialists. The categorization was based on shared ecological requirements and predicted sensitivity to fire-induced habitat alteration (Table 1).

**Table 1:** Mean occupancy (ψ) estimates by landscape zone at baseline (pre-fire month) and 5 months post-fire, categorized by functional group and for the entire community

Functional group	Species	Burned		Proximal		Control	
		*Ψ*(-1)	*Ψ*(5)	*Ψ*(-1)	*Ψ*(5)	*Ψ*(-1)	*Ψ*(5)
COMMUNITY	*n* = 31	0.53	0.33	0.58	0.52	0.45	0.43
Grazers (G)	eland, oryx, red hartebeest, springbok, waterbuck, blue wildebeest, plains zebra	0.35	0.28	0.37	0.39	0.24	0.28
Browsers/Mixed feeders (B)	common duiker, giraffe, kudu, steenbok	0.64	0.5	0.66	0.66	0.56	0.59
Burrow-users (BU)	aardvark, porcupine, scrubhare, springhare, warthog	0.61	0.37	0.66	0.54	0.55	0.51
Large carnivores (LC)	brown hyena, cheetah, leopard	0.49	0.25	0.56	0.47	0.37	0.29
Small carnivore generalists (SCg)	baboon, banded mongoose, cape fox, caracal, honey badger, black-backed jackal, small spotted genet, slender mongoose, African wild cat	0.57	0.32	0.64	0.57	0.54	0.5
Small carnivore specialists (SCs)	aardwolf, bat-eared fox	0.6	0.26	0.66	0.52	0.45	0.31

The distinction between small carnivore generalists and specialists was based on a combination of dietary breadth and habitat: generalists exploit a broad range of food resources across different habitats, whereas specialists depend on narrow, predictable diets, mostly termites and sometimes other invertebrates that tie them more closely to specific microhabitat and substrate conditions (Mills et al. 2019). We acknowledge the fact that browsers encompass diverse body sizes, but were grouped by their shared reliance on more fire-resistant woody vegetation. We further classified brown hyenas (*Parahyaena brunnea*) as large carnivores despite their use of burrows and dens, as their occupancy is driven primarily by landscape-scale mobility and scavenging opportunities rather than localized refuge use characteristic of smaller burrowing species.

### Multi-season multi-species occupancy model

We divided the six-month monitoring period into six primary sampling periods (one month each) and sub-divided each month into three secondary sampling periods (approximately 10 days each) to create species-specific detection histories. This hierarchical structure is the standard for multi-season occupancy models and allows simultaneous estimation of site-level occupancy and detection probability while accounting for imperfect detection.

The multi-season, multi-species occupancy model was implemented in the R package spOccupancy (Doser et al. 2022). This hierarchical Bayesian approach is more powerful and integrative than fitting separate models for each month or running independent occupancy analyses for individual species, as it allows information sharing across species while estimating community and species-specific responses.

We modeled occupancy probability for each species at each site and time period as a function of landscape zones (burned, proximal, control, with burned as the reference), temporal trends across all six months, and landscape-by-time interactions, allowing temporal trends to vary among the three zones. We included the pre-fire baseline in a continuous temporal sequence rather than analyzing pre-fire and post-fire periods separately, as exploratory analyses indicated that temporal trends were similar whether the baseline was included or excluded from the model. We modeled detection probability as a function of baseline detection at tree cameras and road camera effects, allowing detection to vary by camera placement type.

Species-level effects (occupancy and detection parameters) were drawn from community-level normal distributions, allowing information sharing across the 31 species while preserving species-specific heterogeneity. Community-level hyperparameters quantify average effects and among-species variation. Functional group-level effects were derived by averaging posterior estimates of occupancy and detection parameters from all species within each group. To quantify trajectory divergence of functional groups, occupancy contrasts were calculated as differences in occupancy change (occupancy at month 5 minus occupancy at baseline) between pairs of landscape zones (i.e., burned – control, proximal – control, burned – proximal).

We fit the model using Markov Chain Monte Carlo (MCMC) with 3 chains, 200 batches of 25 iterations each (5,000 iterations per chain), a burn-in of 2,000 iterations, and a thinning rate of 12 (yielding 750 posterior samples per parameter). We assessed convergence using the Gelman–Rubin diagnostic ($\:\widehat{R}$ < 1.05) and effective sample size (ESS). Weakly informative priors were specified for all community-level parameters.

## Results

### Post-fire environmental change

After the wildfire, burned areas showed slightly elevated temperatures and consistently lower humidity compared to proximal and control zones (Fig. 1). NDVI trends in proximal and control areas closely aligned and increased with wet season onset, whereas burned-area NDVI declined sharply post-fire before recovering and eventually peaking higher than other zones by month 5.

### Sampling and detection

We recorded 24,387 independent detections of 31 species over six months (September 2021–March 2022). Community-level detection probability was 0.29 (95%CrI: 0.21–0.41) for tree cameras versus 0.47 (95%CrI: 0.33–0.62) for road cameras (Table 2), with species-specific differences (Fig S3).

**Table 2:** Community-level occurrence (ψ) and detection (p) parameters from the multi-species multi-season occupancy model (logit scale), including means, 95% credible intervals, R̂ values and effective sample sizes (ESS)

Parameter	Mean	2.50%	97.50%	R̂	ESS
*Occupancy (ψ)*					
Intercept (burned sites, baseline month)	-0.42	-0.99	0.17	1.03	750
Control (vs. burned)	0.01	-0.33	0.33	1.01	750
Proximal (vs. burned)	0.74	0.44	1.02	1	641
Time	-0.45	-0.66	-0.25	1.01	426
Control x time interaction	0.42	0.19	0.62	1.01	560
Proximal x time interaction	0.32	0.11	0.55	1	421
*Detection (p)*					
Intercept (tree cameras)	-0.88	-1.34	-0.38	1.03	441
Road (vs. tree cameras)	0.78	0.38	1.15	1.04	750

### Community-level occupancy dynamics

**Fig. 2:**
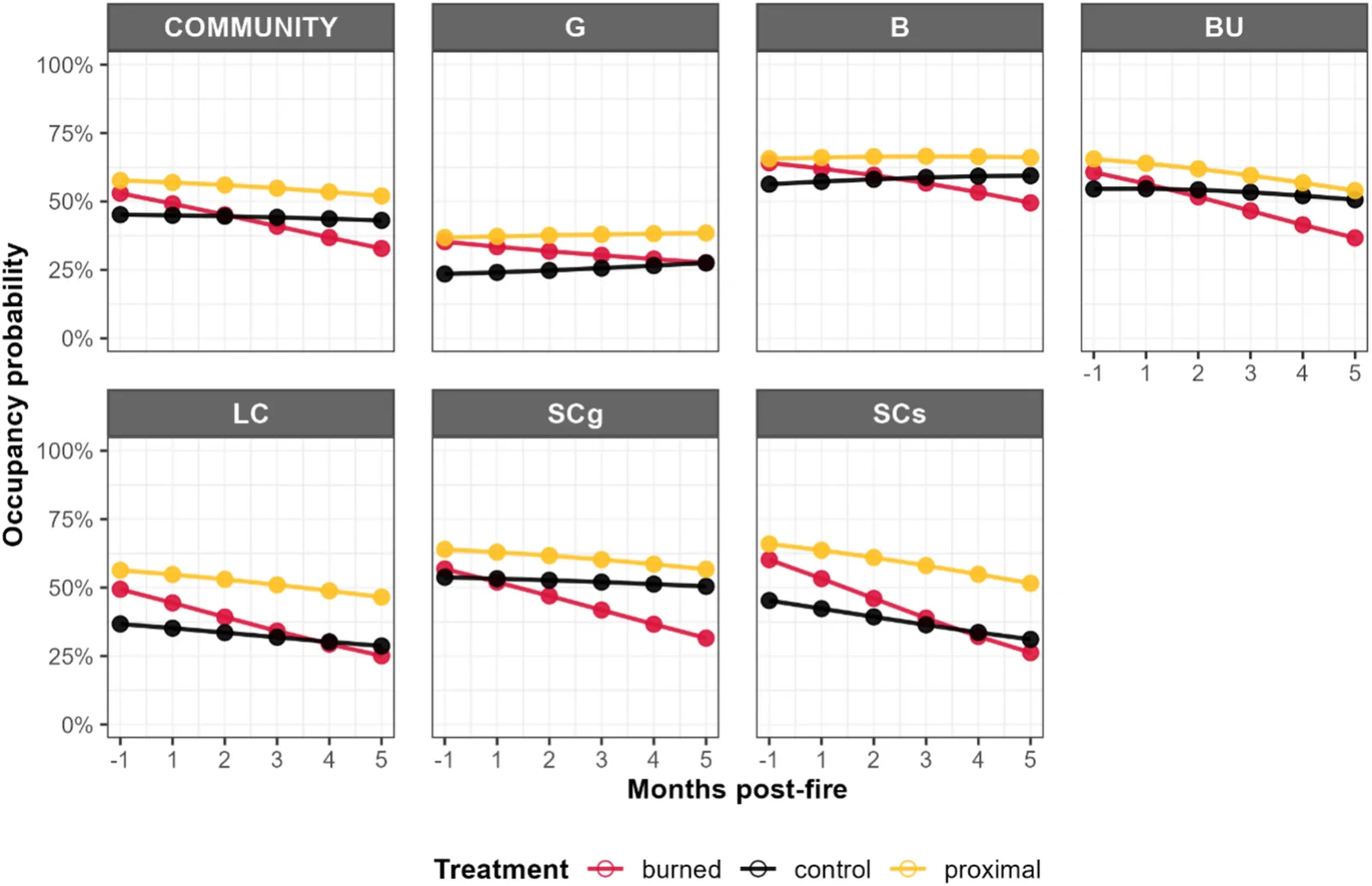
Community- and functional group-level occupancy trends during 6 months (1 month pre-fire baseline and 5 months post-fire) in burned (red), proximal (yellow) and control (black) areas. Community (31 medium- to large mammal species), Grazers (G; *n* = 7), Browsers (B; *n* = 4), Burrow-users (BU; *n* = 5), Large carnivores (LC; *n* = 3), Small carnivore generalists (SCg; *n* = 10), Small carnivore specialists (SCs; *n* = 2)

The multi-species multi-season occupancy model showed a significant difference in community occupancy between landscape zones and with time after the wildfire (Table 2). Baseline occupancy was similar between burned (0.53 (95%CrI: 0.49–0.59)) and control (0.45 (95%CrI: 0.42–0.49)) areas but higher in proximal zones (0.58 (95%CrI: 0.54–0.62)). Temporal interactions confirmed divergent trajectories: burned areas declined to 0.33 (95%CrI: 0.28–0.37) by month 5, control areas remained nearly stable (0.43 (95%CrI: 0.4–0.47)), and proximal areas showed minimal decline (0.52 (95%CrI: 0.48–0.56)) (Fig. 2). MCMC chains converged adequately (all R̂ < 1.1; effective sample sizes: 300–1100 except for waterbuck, cheetah, striped polecat and bat-eared fox, Table S1).

### Functional group dynamics

Mean baseline occupancy varied substantially among functional groups (Fig. 2), with grazers and large carnivores naturally rarer (0.40–0.50) than browsers, burrow-users, and smaller carnivores (0.50–0.70). Temporal responses differed across groups: grazers and browsers showed modest declines in burned areas with slight increases in proximal/control zones, whereas burrow-users and carnivores exhibited steeper declines in burned areas and modest declines elsewhere.

**Fig. 3:**
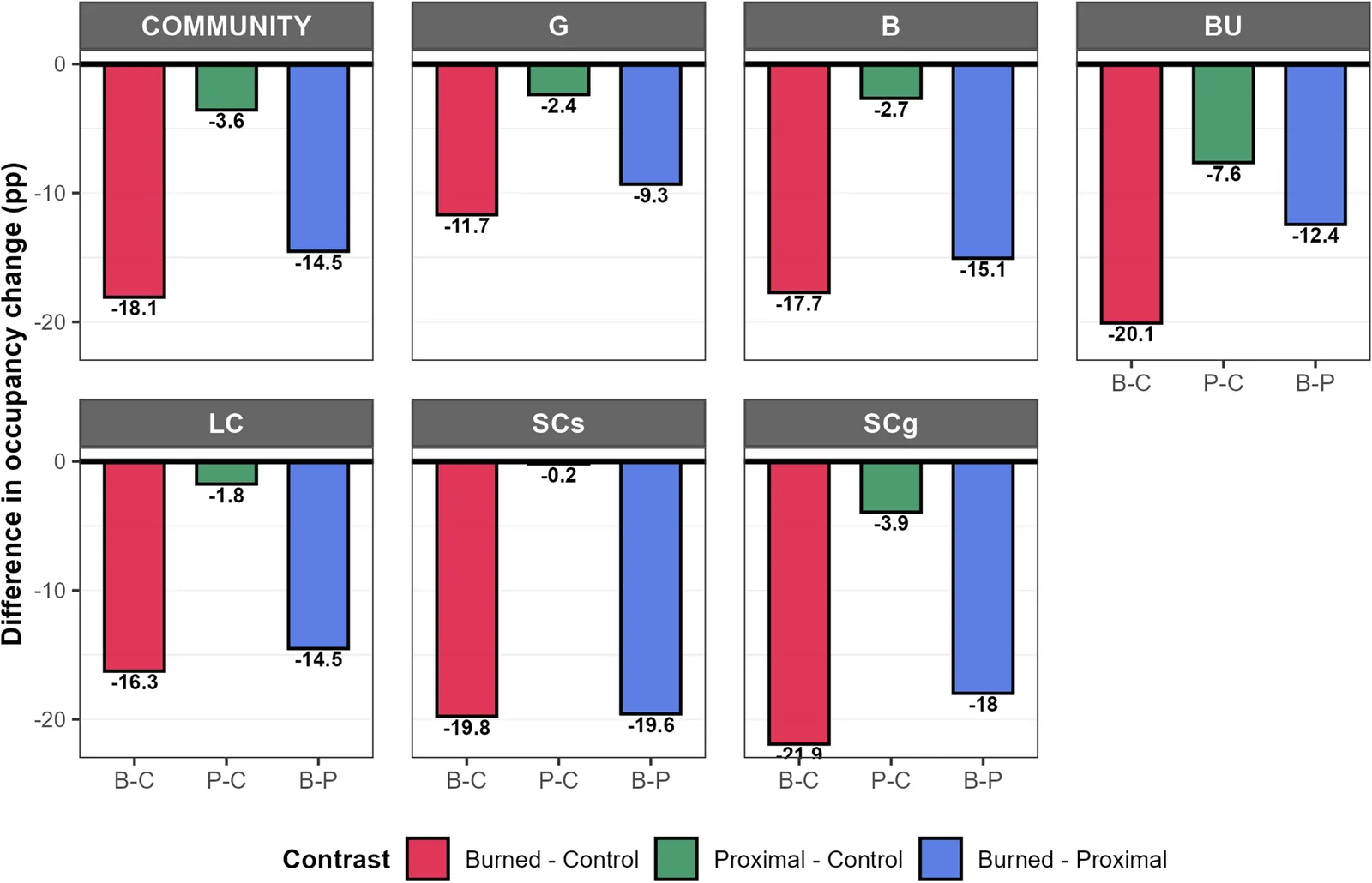
Pairwise contrasts of occupancy change (month 5 - month − 1) between landscape zones: Burned - Control (red), Proximal - Control (green), and Burned - Proximal (blue)

Occupancy contrasts revealed similar fire responses but varied in intensity (Fig. 3). Small carnivore generalists showed the largest occupancy contrast between burned and control areas (− 21.9%), followed by small carnivore specialists (− 19.8%) and burrow-users (− 20.1%). Grazers (− 11.7%) and large carnivores (− 16.3%) showed smaller contrasts. Proximal areas did not show a refuge effect; instead, occupancy diverged negatively from control areas across most groups (− 0.2 to − 7.6%), with burrow-users showing the most pronounced contrast.

### Species-specific temporal responses

**Fig. 4:**
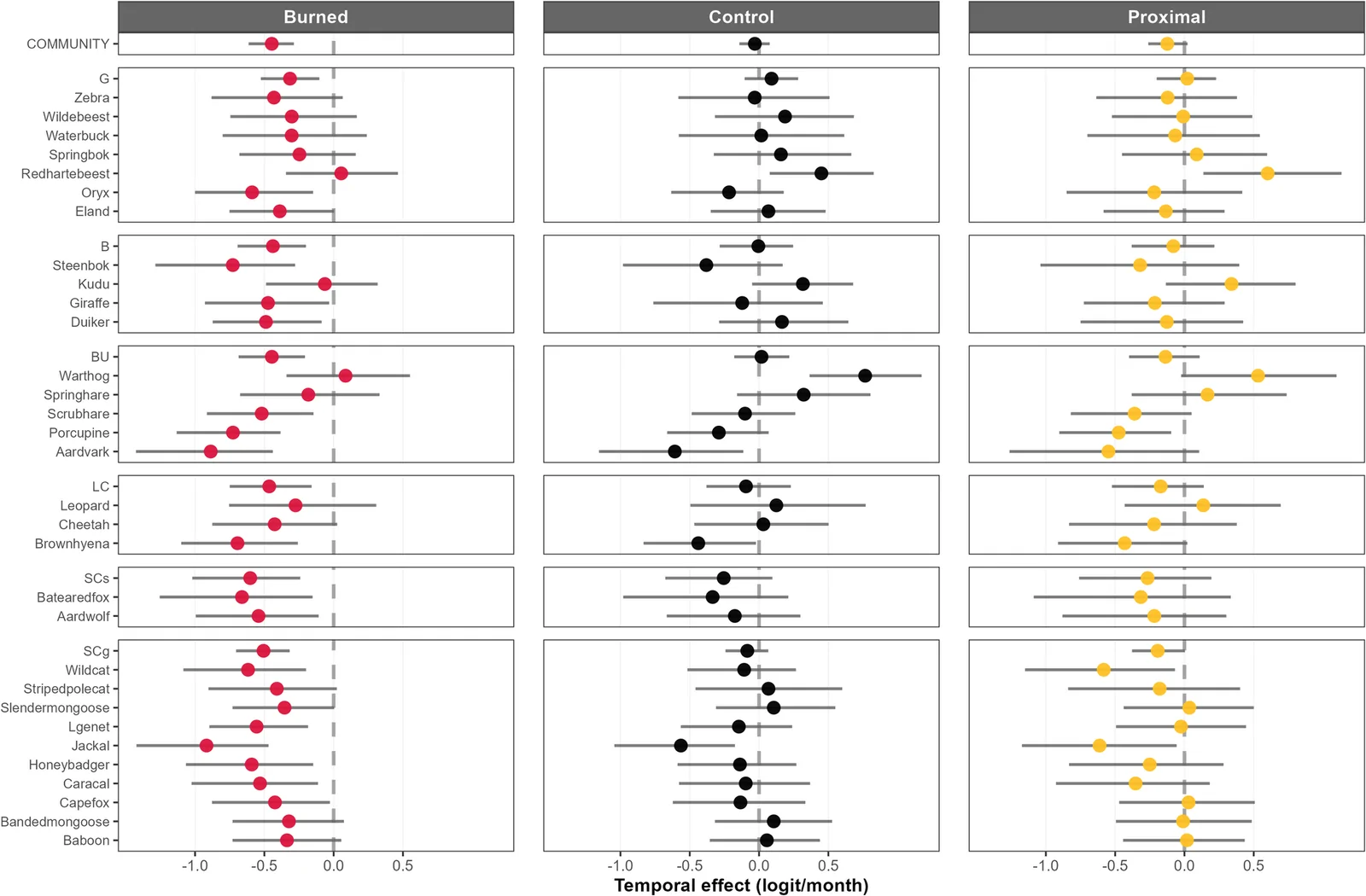
Temporal response coefficients (occupancy change per month, logit scale) for all 31 species organized by functional group (rows) and landscape zone. Each horizontal line represents the 95% credible interval; point shows posterior mean. Negative coefficients indicate occupancy decline over time with a robust effect when 95% credible intervals do not include zero (dashed vertical)

Temporal response coefficients showed species- and area-specific variation in occupancy change rates. The species-specific increases and declines described below are based on effect sizes for which the 95% credible interval did not overlap zero (Fig. 4, Table S2). Species-level occupancy trajectories are presented in Fig S4.

Among grazers, oryx (*Oryx gazella*) and eland (*Taurotragus oryx*) declined in burned areas while remaining stable in control and proximal areas. Red hartebeest (*Alcelaphus buselaphus*) increased in control and proximal areas but remained stable in burned areas. Other grazers showed similar changes between zones, though waterbuck (*Kobus ellipsiprymnus*) showed poor convergence due to limited detections and model outputs for this species should be interpreted cautiously.

Three out of the four browsers and mixed feeder species declined in burned areas while remaining stable in control and proximal areas although giraffe (*Giraffa giraffa*) was overall rare. Only kudu (*Tragelaphus strepsiceros*) showed minimal temporal change in burned areas with a tendency to increase in proximal and control areas.

Among the burrow-dwelling species, porcupine (*Hystrix africaeaustralis*) and scrub hare (*Lepus saxatilis*) declined in burned areas while remaining stable in proximal and control areas. Aardvark (*Orycteropus afer*) declined across both burned and control areas. Warthog (*Phacochoerus africanus*) increased in control and proximal areas but showed no change in burned areas.

Large carnivores displayed small variations between landscape zones: cheetah (*Acinonyx jubatus*) showed modest declines in burned areas, while brown hyena declined across zones. In contrast, in burned zones both small carnivore specialists (aardwolf *Proteles cristatus*, bat-eared fox *Otocyon megalotis*) and most small carnivore generalists showed declines except jackal (*Lupulella mesomelas*; declined across zones), wildcat (*Felis silvestris*; burned/proximal declines), and banded mongoose (*Mungos mungo*) and striped polecat (*Ictonyx striatus*; temporal stability).

## Discussion

Our study demonstrates that large wildfires drive progressive, community-wide declines in mammalian occupancy during the first five months after the fire disturbance. These responses are likely linked to post-fire habitat changes, characterized by elevated temperatures, reduced humidity and suppressed vegetation productivity. Importantly, fire effects extended beyond the burned area itself, reshaping mammal distributions across the broader landscape.

### Post-fire habitat change as driver of mammal occupancy

Post-fire abiotic conditions in burned areas created prolonged thermal stress and reduced structural cover throughout the five-month monitoring period. NDVI declined sharply following the fire and remained lower than in unburned areas until the final month of the study, when it rose to exceed that of unburned areas. This indicates strong vegetation regrowth only late in the wet season, possibly once the burned area had received sufficient rainfall This likely marks the onset of a gradual return of herbivores tracking the fresh, nutrient-rich vegetation (Archibald et al. 2005; Eby et al. 2014), potentially followed by carnivores responding to renewed prey availability (Geary et al. 2020; Gigliotti et al. 2022), and may result in burned areas having a higher occupancy than proximal and control areas. However, capturing this trajectory would require monitoring through the full wet season and into the subsequent dry season, and was beyond the early-phase window addressed in this study.

Measurements from the deployed dataloggers showed only minor temperature differences among zones. However, these were collected on persisting vegetation islands surrounding trees within the burned landscape (Fig S2), where local microclimates may buffer against more extreme thermal conditions of the surrounding matrix (Hoffmann et al. 2012; Liu et al. 2019). The post-fire habitat conditions likely reduced forage availability, prey abundance and shelter for a wide range of species (Doherty et al. 2022). Consequently, the divergent occupancy trajectories between burned and control sites provide evidence that early-phase post-fire habitat changes redistributes mammals progressively following wildfires.

### Functional group responses and trophic constraints

Responses varied among mammalian functional groups, reflecting differences in body size, diet and ecological specialization. Small- to medium-sized carnivores exhibited the strongest fire-driven occupancy declines, and we hypothesize that this relates to reductions in prey following fire (Culhane et al. 2022; Hale et al. 2022), increased exposure and thermoregulatory costs (Liu et al. 2019), loss of fine-scale vegetation structure required for effective hunting (Doherty et al. 2022), and in some cases possibly mortality due to fire. The concurrent declines observed among small burrow-dwelling species like porcupine and scrub hare support the hypothesis that post-fire prey declines contribute to these patterns.

Among larger-bodied species, carnivores showed more modest declines compared to grazers and browsers, which is consistent with their wider ranging behavior and ability to exploit prey across multiple habitat types (Gigliotti et al. 2022). Within herbivores, the response depended on diet as well as size. Contrary to commonly reported post-fire attraction patterns, grazers did not increase occupancy in burned areas, likely because the fire occurred late in the dry season and vegetation recovery lagged behind (Eby et al. 2014). The largest, most grass-dependent species such as oryx and eland, declined most strongly, consistent with their high forage requirements and the loss of grazing resources. On the other hand, species able to draw on alternative and more fire-resilient resources such as red hartebeest, kudu and warthog, maintained stable occupancy in burned areas while increasing in unburned zones.

### Absence of a proximal refuge effect

Contrary to our predictions, proximal areas did not function as refugia for animals displaced from burned areas. Instead, occupancy in proximal zones diverged slightly but consistently below control areas across most functional groups. This finding challenges our displacement–refuge hypothesis and suggests that wildfires generate negative landscape-scale effects beyond the burned zones.

The fire originated in the western portion of the burned area and expanded eastward over multiple days, driven by prevailing wind conditions (Fig. S2). Eastern proximal zones, where camera traps were deployed, possibly experienced prolonged smoke exposure, ash deposition and reduced forage quality (Zahed and Bączek-Kwinta 2023). Western proximal areas remained unmonitored, and occupancy dynamics there may have differed, potentially temporarily concentrating displaced animals. Additionally, displaced wildlife in proximal zones could have intensified predation and competition, offsetting numerical influx from burned areas (Doherty et al. 2022). Because post-fire habitat attributes develop asynchronously across landscapes, fire impacts on fauna can extend well beyond the mapped fire extent, altering habitat suitability and faunal distributions across broader spatial and temporal scales (Haslem et al. 2011). Together, these mechanisms imply that the effective footprint of fires exceeds their burned boundaries, reducing the availability of high-quality refuge habitat.

### Limitations

Our study provides new evidence of wildfire-driven shifts in savanna mammal occupancy, although limitations are acknowledged. Because cattle were removed from the burned areas after the fire, our results reflect the combined effects of both disturbances which we cannot fully separate. The likely effect of removing cattle, however, points away from it being the main driver of our results. Large grazers, and to a lesser extent browsers, may compete with cattle for forage, and tend to avoid cattle spatially and temporally, especially during resource-limited dry periods (Odadi et al. 2011; Kinga et al. 2018). Removing cattle from the burned areas would therefore have relieved this competitive pressure, while we observed persistent declines in burned areas for grazer and browser species, the opposite of what livestock removal alone would predict. Large carnivores could in principle negatively respond to livestock removal through the loss of cattle as an opportunistic prey resource. In our system, however, the only large carnivore that sometimes preys on cattle is the leopard, which was among the least-detected species in our study (*n* = 17 detections) and, across comparable southern African systems, prefers wild prey over livestock (Chase Grey et al. 2017; Jansen et al. 2023). Livestock removal is therefore unlikely to have substantially altered the prey base of large carnivores, and their declines in burned areas are better explained by fire-driven loss of habitat structure, cover, and wild prey.

Additional limitations include that we did not quantify spatial variation in fire intensity or severity, which can strongly influence vegetation recovery, microclimates, and resource availability within burned areas, and that wildlife responses may differ for fires occurring at other times of the year. We derived functional group trajectories from species-level estimates, simplifying ecological diversity but revealing consistent patterns. Additionally, we summarized model outputs without propagating uncertainty into group-level inference, though uncertainty was represented and visualized at the species level. Our camera trap network was designed for medium- to large-bodied mammals, underrepresenting and omitting smaller taxa. Despite these caveats, the consistent patterns across groups and spatial zones indicated substantial, landscape-scale impacts of the wildfire.

### Implications for savanna fire ecology and conservation

Our findings underscore the significance of early post-fire dynamics in shaping mammal community composition and distribution across savanna landscapes. The observed declines during the initial months post-fire suggest that repeated exposure to such conditions could threaten population persistence, particularly for small-bodied and specialist taxa. Conservation strategies that maintain large landscapes with spatial heterogeneity and connectivity could provide access to unburned refuges and facilitate during and post-fire movements, thereby helping buffer wildlife communities from fire effects. Integrating telemetry tags and fine-scale behavioral and physiological data would further aid in understanding how species redistribute and recover in landscapes highly susceptible to wildfires.

## Supplementary Information

Below is the link to the electronic supplementary material.

Supplementary Material 1

## Data Availability

Data will be made available upon reasonable request.
