# Supplementary material for: Early post-fire declines of mammals in a tree-and-shrub savanna
Source: Oecologia. 2026 Jul 23;208(8):100. doi: 10.1007/s00442-026-05939-w (PMC13396034; doi:10.1007/s00442-026-05939-w)
Supplement: Supplementary file 1 — Supplementary Material 1 [file 442_2026_5939_MOESM1_ESM.docx]

**Electronic Supplementary Material for:** Early post-fire declines of mammals in a tree-and-shrub savanna

Stijn Verschueren^1,2*^, Maria Laura Ruozzi^1,3^, Laurie Marker^1^, Herwig Leirs^3^ & Bogdan Cristescu^1,4,5^

^1^Cheetah Conservation Fund, Otjiwarongo, Namibia

^2^Division of Conservation Biology, University of Bern, Bern, Switzerland

^4^School of Agriculture and Natural Resources Sciences, Namibia University of Science and Technology, Windhoek, Namibia

^5^School of Applied Sciences, University of Brighton, Brighton, United Kingdom

*Corresponding author: [stijn@cheetah.org](mailto:stijn@cheetah.org)


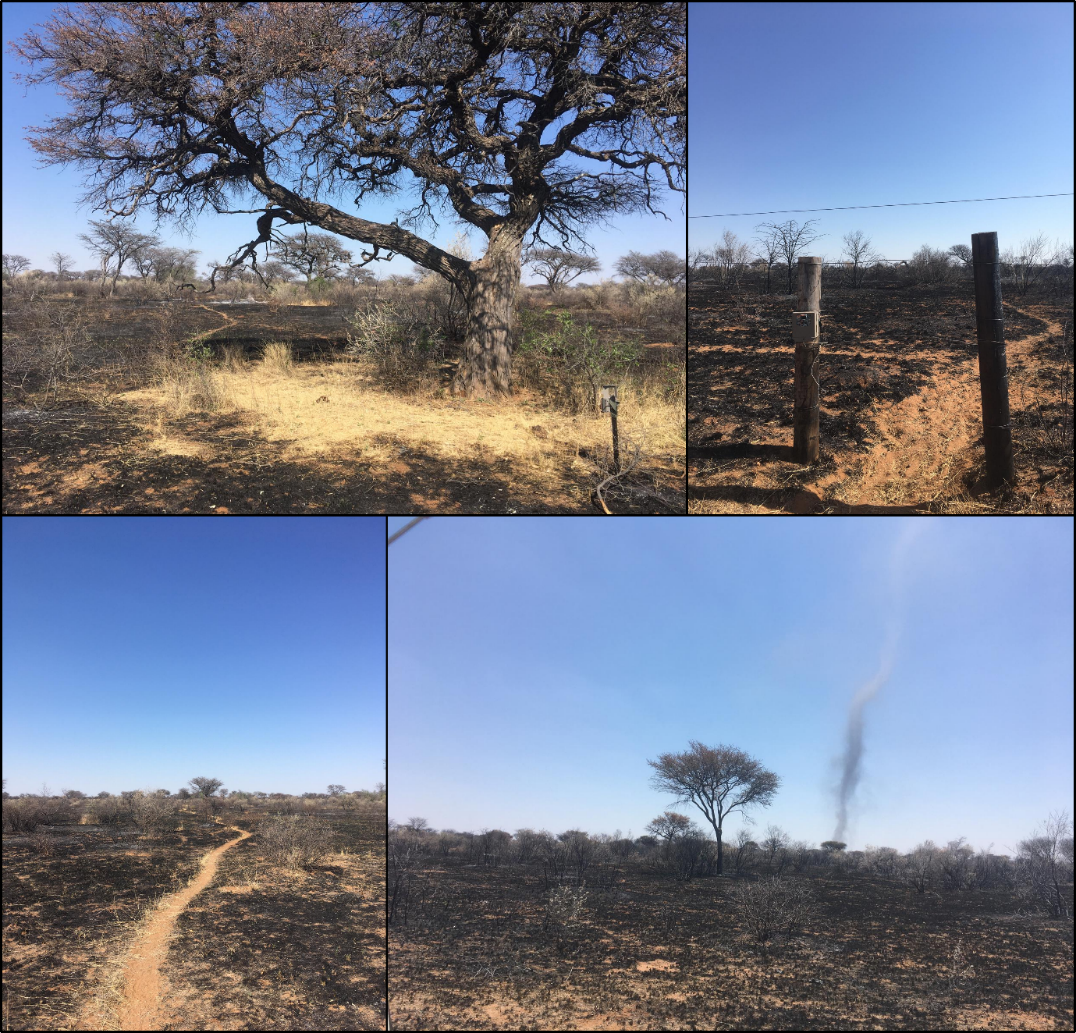


**Figure S1.** Images from the burned area (13 October 2021), illustrating postfire conditions and camera trap deployment. Top left: Large tree with surrounding vegetation persisting post-fire, providing a buffering microclimate and monitored by camera traps and dataloggers. Top right: Camera trap at road-wildlife trail intersection (road deployment). Bottom left: Wildlife trail through burned landscape. Bottom right: Dust devil redistributing ash.


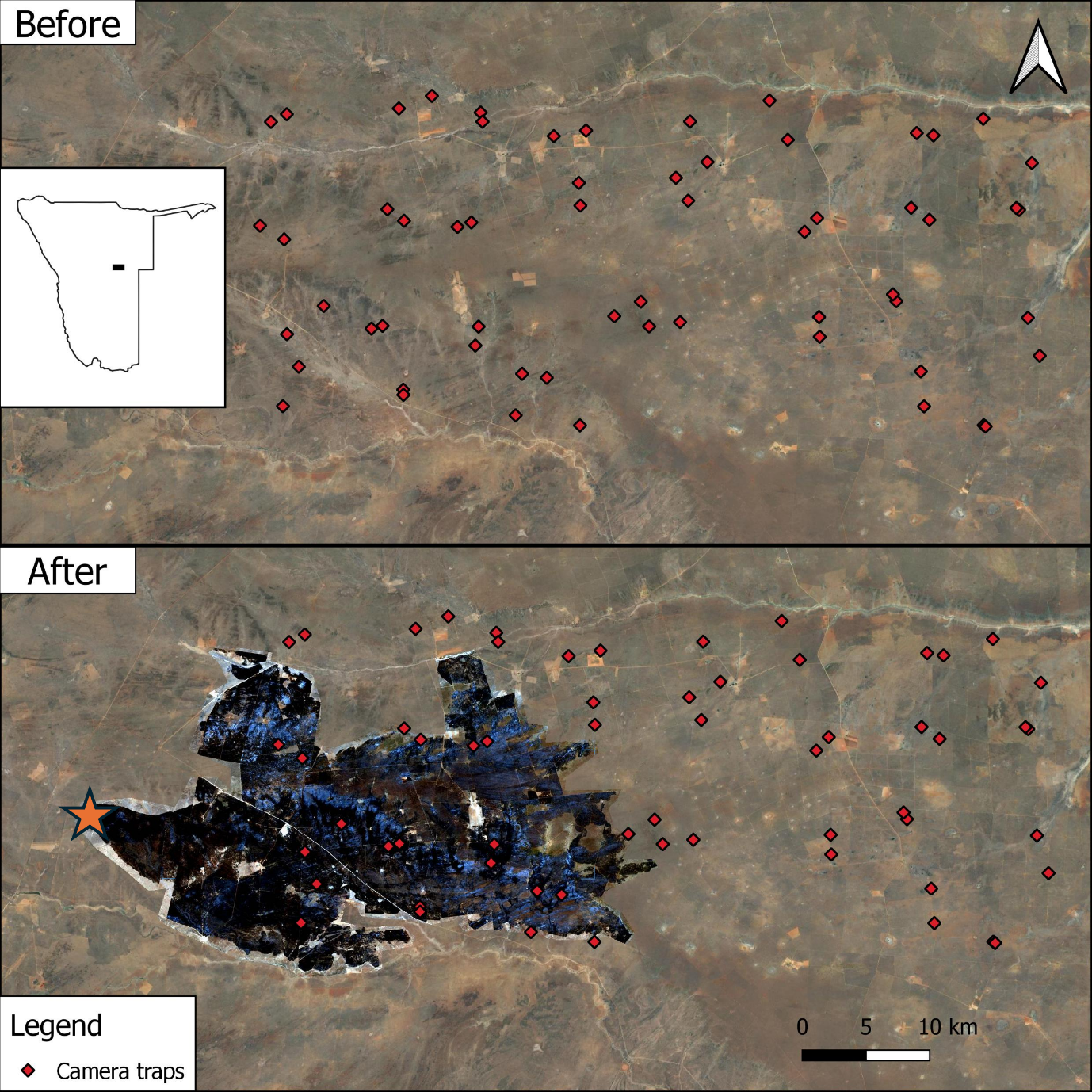


**Figure S2.** Satellite images of the study area one week before and after the wildfire. The fire originated in the western section (orange star) and expanded eastwards following the prevailing wind conditions.


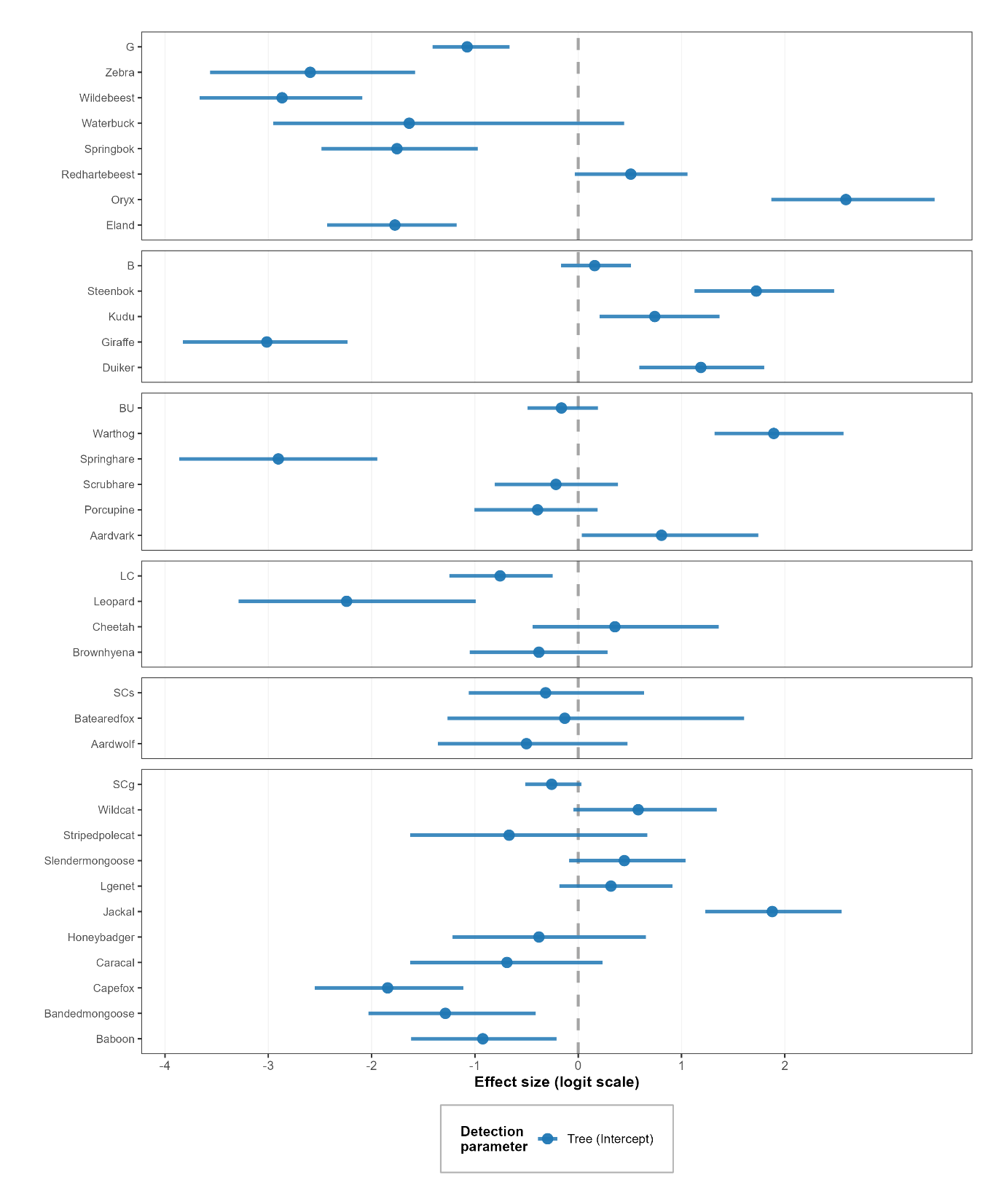


**Figure S3.** Detection probability estimates for tree versus road camera traps for all 31 species organized by functional group. Horizontal lines = 95% credible intervals; points = posterior means. Grazers (G; n=7), Browsers (B; n=4), Burrow-users (BU; n=5), Large carnivores (LC; n=3), Small carnivore specialists (SCs; n=2), Small carnivore generalists (SCg; n=10).


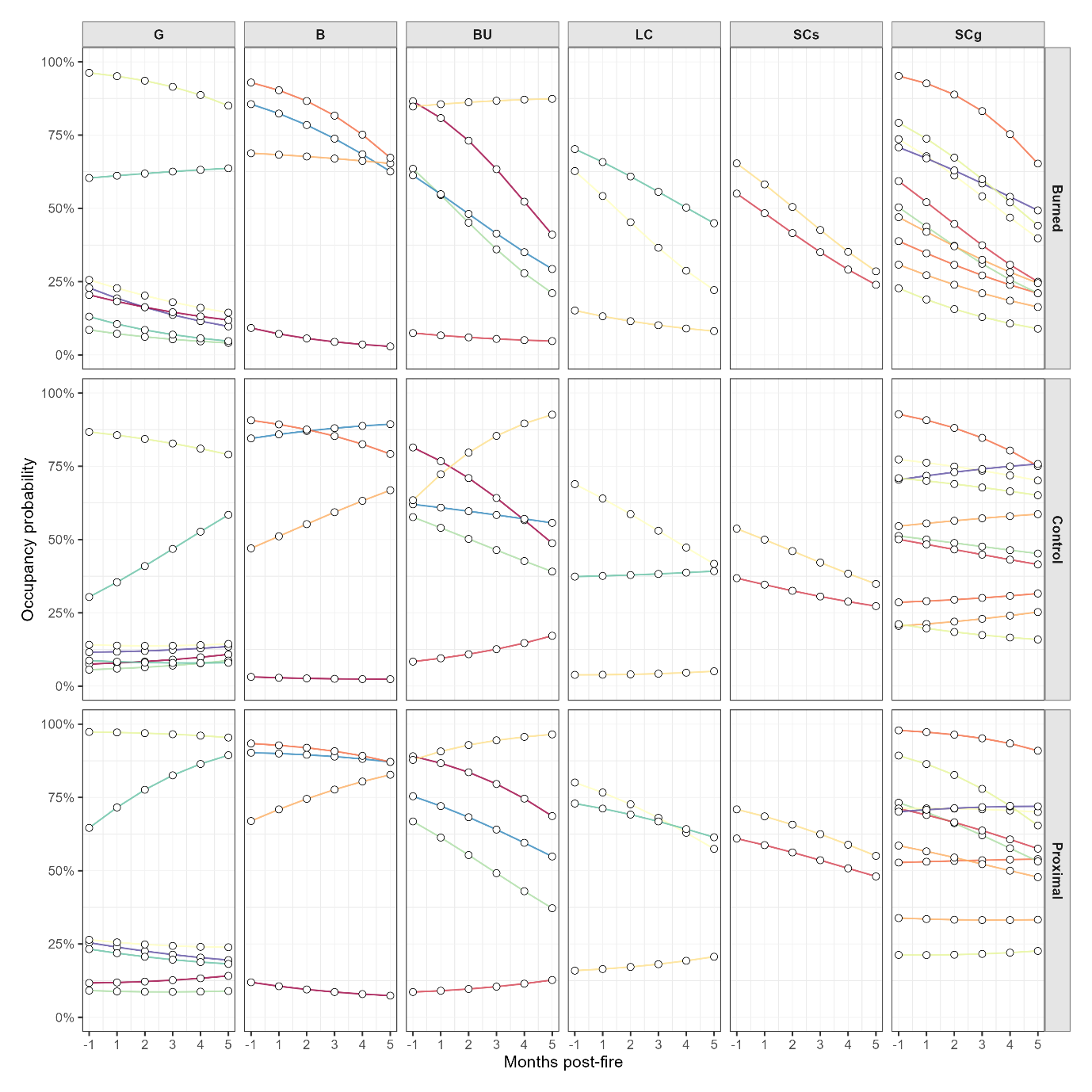


**Figure S4.** Species-level occupancy trajectories during 6 months (1 month pre-fire baseline and 5 months post-fire) in burned, proximal and control areas. Grazers (G; n=7), Browsers (B; n=4), Burrow-users (BU; n=5), Large carnivores (LC; n=3), Small carnivore specialists (SCs; n=2), Small carnivore generalists (SCg; n=10). Individual species legends are omitted for clarity due to the high number of species and color codes. Please see Figure 4 in the main manuscript for species-specific responses and identification.

**Table S1.** Number of independent detections (>30min) of 31 mammalian wildlife species (>1kg) recorded in burned, control and proximal areas. Model convergence diagnostics are presented by the Gelman–Rubin factor (R̂) and the effective sample size (ESS). Grazers (G; n=7), Browsers (B; n=4), Burrow-users (BU; n=5), Large carnivores (LC; n=3), Small carnivore generalists (SCg; n=10), Small carnivore specialists (SCs; n=2).

| **Group** | **Species** | **Burned** | **Control** | **Proximal** | **R̂** | **ESS** |
| --- | --- | --- | --- | --- | --- | --- |
| G | Eland | 30 | 47 | 255 | 1.014 | 750 |
|  | Oryx | 1555 | 1635 | 2311 | 1.006 | 750 |
|  | Red hartebeest | 642 | 116 | 313 | 1.027 | 750 |
|  | Springbok | 145 | 2 | 6 | 1.003 | 775 |
|  | Waterbuck | 15 | 0 | 12 | 1.096 | 91 |
|  | Wildebeest | 6 | 43 | 35 | 1.025 | 750 |
|  | Zebra | 0 | 11 | 39 | 1.016 | 411 |
| B | Giraffe | 22 | 6 | 245 | 1.002 | 750 |
|  | Duiker | 270 | 574 | 661 | 1.012 | 750 |
|  | Steenbok | 236 | 414 | 444 | 1.011 | 750 |
|  | Kudu | 104 | 161 | 233 | 1.003 | 750 |
| BU | Aardvark | 45 | 132 | 121 | 1.059 | 599 |
|  | Porcupine | 46 | 119 | 94 | 1.012 | 704 |
|  | Scrubhare | 221 | 542 | 395 | 1.021 | 371 |
|  | Springhare | 1 | 25 | 24 | 1.021 | 600 |
|  | Warthog | 905 | 1321 | 1448 | 1.02 | 843 |
| LC | Brown hyena | 35 | 127 | 110 | 1.014 | 864 |
|  | Cheetah | 53 | 32 | 47 | 1.024 | 271 |
|  | Leopard | 5 | 0 | 12 | 1.02 | 337 |
| SCg | Baboon | 19 | 43 | 98 | 1.025 | 616 |
|  | Banded mongoose | 12 | 35 | 60 | 1 | 471 |
|  | Cape fox | 21 | 55 | 35 | 1.013 | 750 |
|  | Caracal | 5 | 59 | 61 | 1.024 | 552 |
|  | Honey badger | 13 | 37 | 54 | 1.011 | 393 |
|  | Jackal | 927 | 1760 | 2611 | 1.002 | 1028 |
|  | Lgenet | 166 | 288 | 165 | 1.011 | 1104 |
|  | Slender mongoose | 203 | 379 | 176 | 1.034 | 750 |
|  | Striped polecat | 1 | 50 | 66 | 1.058 | 260 |
|  | Wild cat | 52 | 176 | 155 | 1.002 | 750 |
| SCs | Aardwolf | 21 | 26 | 49 | 1.035 | 450 |
|  | Bat-eared fox | 11 | 20 | 30 | 1.163 | 113 |

**Table S2.** Species-level occupancy parameters from the multi-species multi-season occupancy model (logit scale), including means and 95% confidence intervals. Grazers (G; n=7), Browsers (B; n=4), Burrow-users (BU; n=5), Large carnivores (LC; n=3), Small carnivore generalists (SCg; n=10), Small carnivore specialists (SCs; n=2).

| **Group** | **Species** | **Burned area occupancy** | | | **Control effect** | | | **Proximal effect** | | | **Temporal trend** | | |
| --- | --- | --- | --- | --- | --- | --- | --- | --- | --- | --- | --- | --- | --- |
|  |  | Mean | CI_2.5 | CI_97.5 | Mean | CI_2.5 | CI_97.5 | Mean | CI_2.5 | CI_97.5 | Mean | CI_2.5 | CI_97.5 |
| G | Eland | -1.774 | -2.429 | -1.177 | -0.231 | -0.991 | 0.486 | 0.486 | -0.194 | 1.159 | -0.389 | -0.752 | 0.003 |
|  | Oryx | 2.591 | 1.869 | 3.450 | -0.944 | -1.922 | -0.184 | 0.903 | 0.019 | 1.898 | -0.589 | -1.000 | -0.148 |
|  | Redhartebeest | 0.509 | -0.034 | 1.058 | -0.759 | -1.428 | -0.083 | 0.919 | 0.273 | 1.662 | 0.055 | -0.344 | 0.464 |
|  | Springbok | -1.755 | -2.485 | -0.972 | -0.649 | -1.506 | 0.208 | -0.246 | -1.126 | 0.547 | -0.247 | -0.680 | 0.159 |
|  | Waterbuck | -1.636 | -2.952 | 0.444 | -0.458 | -1.566 | 0.635 | 0.360 | -0.510 | 1.278 | -0.303 | -0.801 | 0.239 |
|  | Wildebeest | -2.867 | -3.665 | -2.090 | 0.185 | -0.760 | 1.069 | 0.465 | -0.438 | 1.288 | -0.302 | -0.746 | 0.167 |
|  | Zebra | -2.595 | -3.564 | -1.579 | 0.040 | -0.986 | 1.039 | 1.155 | 0.384 | 2.055 | -0.431 | -0.882 | 0.065 |
| B | Duiker | 1.187 | 0.592 | 1.801 | 0.795 | -0.006 | 1.614 | 0.980 | 0.278 | 1.799 | -0.489 | -0.873 | -0.087 |
|  | Giraffe | -3.015 | -3.827 | -2.234 | -0.738 | -1.876 | 0.292 | 0.661 | -0.178 | 1.460 | -0.474 | -0.929 | -0.033 |
|  | Kudu | 0.741 | 0.207 | 1.368 | -0.442 | -1.109 | 0.215 | 0.455 | -0.284 | 1.128 | -0.064 | -0.488 | 0.317 |
|  | Steenbok | 1.724 | 1.126 | 2.477 | 0.186 | -0.626 | 1.059 | 0.729 | -0.079 | 1.580 | -0.728 | -1.286 | -0.278 |
| BU | Aardvark | 0.806 | 0.035 | 1.743 | -0.044 | -0.810 | 0.737 | 0.755 | -0.006 | 1.561 | -0.887 | -1.427 | -0.439 |
|  | Porcupine | -0.393 | -1.004 | 0.189 | 0.325 | -0.302 | 1.017 | 0.485 | -0.198 | 1.105 | -0.727 | -1.133 | -0.383 |
|  | Scrubhare | -0.216 | -0.808 | 0.385 | 0.586 | -0.087 | 1.233 | 0.898 | 0.298 | 1.519 | -0.519 | -0.915 | -0.145 |
|  | Springhare | -2.903 | -3.863 | -1.946 | 0.819 | -0.092 | 1.820 | 0.634 | -0.225 | 1.501 | -0.183 | -0.675 | 0.331 |
|  | Warthog | 1.893 | 1.319 | 2.569 | -0.310 | -1.105 | 0.432 | 0.876 | 0.148 | 1.686 | 0.086 | -0.340 | 0.550 |
| LC | Brownhyena | -0.381 | -1.050 | 0.283 | 0.621 | -0.142 | 1.391 | 1.272 | 0.539 | 2.095 | -0.694 | -1.100 | -0.258 |
|  | Cheetah | 0.355 | -0.440 | 1.360 | -0.857 | -1.782 | 0.018 | 0.470 | -0.440 | 1.313 | -0.426 | -0.876 | 0.026 |
|  | Leopard | -2.242 | -3.288 | -0.993 | -1.140 | -2.466 | -0.068 | 0.591 | -0.382 | 1.399 | -0.275 | -0.755 | 0.308 |
| SCg | Baboon | -0.924 | -1.618 | -0.210 | 0.049 | -0.663 | 0.716 | 1.070 | 0.394 | 1.807 | -0.337 | -0.730 | 0.055 |
|  | Bandedmongoose | -1.286 | -2.031 | -0.411 | 0.011 | -0.763 | 0.741 | 0.562 | -0.225 | 1.277 | -0.323 | -0.730 | 0.074 |
|  | Capefox | -1.845 | -2.549 | -1.113 | 0.292 | -0.539 | 1.061 | 0.518 | -0.180 | 1.211 | -0.424 | -0.878 | -0.027 |
|  | Caracal | -0.690 | -1.626 | 0.235 | 0.619 | -0.129 | 1.590 | 1.302 | 0.572 | 2.240 | -0.531 | -1.025 | -0.113 |
|  | Honeybadger | -0.380 | -1.217 | 0.655 | 0.205 | -0.638 | 1.008 | 1.050 | 0.314 | 1.960 | -0.591 | -1.066 | -0.148 |
|  | Jackal | 1.879 | 1.229 | 2.551 | 0.003 | -0.759 | 0.784 | 1.353 | 0.556 | 2.402 | -0.918 | -1.423 | -0.470 |
|  | Genet | 0.317 | -0.182 | 0.914 | 0.760 | 0.146 | 1.488 | 0.598 | -0.022 | 1.234 | -0.556 | -0.897 | -0.184 |
|  | Slendermongoose | 0.446 | -0.087 | 1.038 | 0.598 | -0.050 | 1.287 | 0.498 | -0.193 | 1.205 | -0.355 | -0.730 | 0.004 |
|  | Stripedpolecat | -0.669 | -1.627 | 0.669 | 0.968 | 0.064 | 1.991 | 0.825 | 0.008 | 1.614 | -0.410 | -0.903 | 0.023 |
|  | Wildcat | 0.581 | -0.048 | 1.341 | 0.205 | -0.574 | 0.904 | 0.871 | 0.198 | 1.604 | -0.618 | -1.084 | -0.199 |
| SCs | Aardwolf | -0.501 | -1.357 | 0.478 | -0.302 | -1.110 | 0.459 | 0.716 | 0.052 | 1.511 | -0.542 | -0.995 | -0.109 |
|  | Batearedfox | -0.130 | -1.265 | 1.606 | -0.106 | -1.127 | 0.921 | 0.815 | -0.032 | 1.768 | -0.662 | -1.255 | -0.152 |
